# Special Issue: State-of-the-Art Nanophotonic and Optical Nanomaterials in China

**DOI:** 10.3390/nano12132270

**Published:** 2022-07-01

**Authors:** Kun Huang, Heping Zeng

**Affiliations:** 1 State Key Laboratory of Precision Spectroscopy, East China Normal University, Shanghai 200062, China; 2 Chongqing Key Laboratory of Precision Optics, Chongqing Institute of East China Normal University, Chongqing 401121, China

In recent years, the fields of nanophotonics and nano-optics have been greatly fueled by rapid advancements in photonic science and technology, especially by the emergence of novel optical nanomaterials and new functional nanostructures. Indeed, state-of-the-art nanophotonic platforms that can precisely control light in subwavelength volumes and thus significantly enhance light-matter interactions have enabled the unraveling of unprecedented optical phenomena in multidisciplinary fields among metamaterials, photonic crystals, and plasmonics. In parallel, new scientific breakthroughs have translated into technological advances, which, in turn, instigate explosive growth in wide-ranging applications, such as ultrasensitive biochemical sensing, highly efficient solar cells, miniaturized spectrometer, super-resolution microscopy, nanoelectronics, and nanofabrication.

This Special Issue aims to provide an overview of the current advances in nanophotonic and optical nanomaterials in China and to prompt new interest in this field. The collected eight articles [1,2,3,4,5,6,7,8] highlight the latest progress in nanophotonics and materials science, including developments of novel nanomaterials and ingenious nanoscale designs. These contributions may unveil new opportunities to control light, provide effective ways to optimize targeted performances, and have important implications for novel photonic devices operating beyond the diffraction limit.

Nanomaterials have unique mechanical, electronic, and optical properties due to the strong confinement of electrons, photons, and phonons at the nanoscale. Particularly, nanoporous gold (NPG) was used by Cui et al. to investigate the enhancement of fluorescence resonance energy transfer (FRET) based on polyelectrolyte multilayers as dielectric spacers between fluorescent protein pairs and plasmonic nanostructures [1], which resulted in a significant improvement on the fluorescence intensity and FRET efficiency. In a study by Kong et al., cadmium telluride (CdTe) was assembled on an AuAg alloy substrate to obtain a fivefold enhancement in terahertz emission compared with that based on silicon substrate [2], thus providing an effective way to produce small, thin, and efficient terahertz photonic devices. Notably, nanomaterial structures play important roles in the optimization of device performances. In this context, vertically aligned Au nanorod arrays with two different diameters were prepared by Ma et al. based on the evaporation self-assembly method, which was used to realize a strong plasmon enhancement on the second harmonic generation [3]. Yu et al. proposed a novel method to prepare high dispersibility silver nano-ink-filled particles, in which the addition of filling particles could effectively improve its electrical conductivity without significantly changing the metal solid content in the ink [4]. Another important direction is to implement functional optoelectronic devices based on advanced fabrication technologies. Specifically, Liu et al. designed and fabricated an ultracompact broadband-polarizing beam splitter based on a tilted nanograting waveguide structure [5], showing a wide operation bandwidth with a favorable tolerance to manufacturing deviations. Additionally, Zeng et al. focused on characterizing and optimizing silicon Mach–Zehnder modulators based on a comprehensive theoretical model [6]. The presented devices would potentially be useful in silicon photonics and related communication networks. Finally, two studies are presented in this collection to highlight promising applications in photocatalysts and solar cells, respectively. Hydrogenated amorphous titania with engineered surface oxygen vacancy was successfully prepared by Feng et al. based on the liquid plasma-induced hydrogenation strategy [7], which provided a promising way to fabricate hydrogenated amorphous photocatalysts and may facilitate visible-light-driven photocatalytic environmental applications. Zhao et al. implemented thin-film solar cells based on a selenized CuSbS_2_ absorber [8], which resulted in increased efficiency of photovoltaic devices to meet the requirement in large-scale applications.

The above articles in this Special Issue present some of the challenges and opportunities in the field of nanophotonics and nanomaterials, which are expected to have a broad impact on a variety of applications in areas of scientific, industrial, and societal significance. We appreciate the efforts and contributions of all authors and hope that this Special Issue will be of broad interest to researchers working in the rapidly expanding field of optics, plasmonics, and nanophotonics.
